# Nurse Migration and Workforce Dynamics in the Eastern Mediterranean Region

**DOI:** 10.1177/23779608261479007

**Published:** 2026-08-10

**Authors:** Abdulqadir J. Nashwan, Salam Bani Hani, Lamiaa Abd El Hakeem Ali Ahmed, Manal Saleh Moustafa Saleh, Abdelaziz Hendy, Jibin Kunjavara, Majeda M. El-Banna

**Affiliations:** 1 Nursing and Midwifery Research Department, 36977 Hamad Medical Corporation, Doha, Qatar; 2 Nursing Department, Irbid National University, Irbid, Jordan; 3 Department of Psychiatric Mental Health Nursing, Faculty of Nursing, 63526 Cairo University, Cairo, Egypt; 4 Department of Mental Health Nursing, North Private College of Nursing, Arar, Saudi Arabia; 5 Nursing Administration, Faculty of Nursing, 68799 Zagazig University, Zagazig, Egypt; 6 Department of Nursing Sciences, College of Applied Medical Science, Shaqra University, Shaqra, Saudi Arabia; 7 Department of Pediatric Nursing, Faculty of Nursing, 496067 Ain Shams University, Cairo, Egypt; 8 Department of Maternal and Child Health Nursing, College of Nursing, Qassim University, Buraydah, Saudi Arabia; 9 College of Nursing, Health Sector, 658148 Qatar University, Doha, Qatar

**Keywords:** nurse migration, eastern mediterranean region, health workforce policy, ethical recruitment, workforce nationalization

## Abstract

Nurse migration is a defining feature of health workforce dynamics in the Eastern Mediterranean Region (EMR), which functions as both a source and destination for internationally mobile nurses. Persistent workforce shortages, increasing healthcare demands, demographic changes, and sociopolitical instability in some countries have intensified cross-border mobility, raising concerns about workforce sustainability and equitable access to nursing care. This paper examines evolving patterns of nurse migration in the EMR and identifies policy priorities to support a sustainable nursing workforce. Drawing on findings from a recent global nurse migration report and relevant regional and international literature, the paper critically synthesizes evidence on migration trends, workforce distribution, and policy responses, with particular attention to governance, nursing education, and recruitment practices. The review highlights contrasting migration patterns across the region. Several middle-income countries continue to experience significant outward migration driven by economic opportunities, career advancement, and working conditions, whereas high-income countries rely extensively on internationally educated nurses to address workforce shortages and support health system expansion. Although initiatives promoting ethical international recruitment, workforce monitoring, and national workforce development are increasing, important challenges remain, including fragmented governance, inconsistent regulatory frameworks, limited educational capacity, sociocultural barriers to nursing careers, and difficulties retaining experienced nurses. Strengthening workforce sustainability in the EMR will require coordinated national and regional workforce planning, greater investment in nursing education, effective retention strategies, enforcement of ethical recruitment practices, and enhanced regional collaboration. These measures can strengthen local nursing capacity, improve workforce stability, and support resilient health systems capable of delivering high-quality, equitable patient care.

## Introduction

The 2024 TruMerit™ Nurse Migration Report highlights a pivotal moment for the Eastern Mediterranean Region (EMR), which simultaneously functions as both a major source and destination of the global nursing workforce (Shaffer & Álvarez, 2024). This dual role creates complex workforce challenges, as persistent nurse shortages, economic disparities, and sociopolitical instability continue to drive migration from several EMR countries, while Gulf Cooperation Council (GCC) countries increasingly rely on internationally educated nurses to sustain expanding healthcare systems (Chirau et al., 2024; Kingma, 2018; Okyay et al., 2021; TruMerit, 2024). Although international migration provides valuable professional and economic opportunities for nurses, excessive dependence on migrant nurses may exacerbate workforce inequities and weaken health systems in source countries (Boldbaatar, 2020; Hani & Nashwan, 2024). This commentary discusses the key drivers of nurse migration in the EMR, examines current policy responses, and highlights priorities for developing a sustainable and ethically governed nursing workforce.

## Brief Review/Discussion of the Topic

Nurse migration within the EMR is influenced by multiple interconnected push and pull factors. Continued emigration from countries such as Iran, Lebanon, Jordan, Egypt, and Pakistan reflects persistent health system challenges, including inadequate remuneration, excessive workloads, limited career advancement, poor professional recognition, and job dissatisfaction (Mohtashami et al., 2025; Nezhad et al., 2025). In addition, workforce shortages, insufficient educational capacity, and constrained professional development opportunities continue to undermine both quality of care and workforce retention (Khazaei et al., 2025). Consequently, addressing these structural challenges is likely to provide a more sustainable workforce solution than continued dependence on international recruitment.

At the same time, demographic and epidemiological transitions have intensified the demand for nursing services across the region. Population ageing, the increasing burden of non-communicable diseases, and the long-term effects of the COVID-19 pandemic have substantially increased healthcare workforce requirements (Nashwan et al., 2021). Several Gulf countries have strengthened international recruitment pathways to address these demands; however, implementation of ethical recruitment practices remains inconsistent, particularly regarding recruitment agency oversight and protection of migrant nurses’ rights (Nashwan et al., 2021; Shaffer et al., 2022).

## Current Insights and Interpretations

Recent evidence suggests that long-term workforce sustainability cannot be achieved through international recruitment alone. Nationalization initiatives across GCC countries represent important investments in strengthening domestic nursing capacity, although barriers including societal perceptions of nursing, limited educational capacity, and retention challenges continue to impede progress (Albejaidi & Nair, 2021; Elbanna et al., 2024). Qatar has expanded scholarship opportunities, strengthened nursing education programmes, and promoted greater public awareness of the nursing profession; nevertheless, the continued reliance on expatriate nurses indicates that workforce self-sufficiency remains a long-term objective rather than an immediate reality (Elsharnouby et al., 2024). Similar experiences across the Gulf reinforce the importance of integrated strategies encompassing education, recruitment, retention, and workforce planning (World Health Organization [WHO], 2020).

Beyond the Gulf region, bilateral migration agreements between North African countries and Europe, together with Pakistan’s labour export strategy, generate important economic benefits through remittances but may also reduce domestic workforce capacity when migration is not strategically managed (Abderrahim, 2024; Khowaja-Punjwani, 2020). These findings highlight the need for regional workforce governance that balances national health workforce requirements with nurses’ legitimate right to international professional mobility.

## Conclusions and Importance to the Nursing Profession

The evidence indicates that nurse migration in the EMR should be viewed as a regional workforce governance issue rather than solely an international recruitment challenge. Future workforce policies should prioritize improving working conditions, remuneration, professional recognition, and career development opportunities to strengthen nurse retention in source countries ([Bibr bibr20-23779608261479007][Bibr bibr4-23779608261479007][Bibr bibr14-23779608261479007]). Simultaneously, Gulf countries should continue investing in domestic nursing education while progressively reducing dependence on expatriate nurses through sustainable workforce planning (WHO, 2025). Stronger implementation of ethical recruitment standards, harmonized regional workforce data, collaborative policy development, and circular migration mechanisms will be essential to achieve equitable workforce distribution (Shaffer et al., 2022; WHO, 2023). Furthermore, gender-responsive workforce policies, including flexible working arrangements and protection from workplace harassment, remain critical given the predominantly female nursing workforce (Elbanna et al., 2024). Collectively, these strategies can strengthen workforce resilience, promote ethical international mobility, and support sustainable health systems across the Eastern Mediterranean Region (Shaffer & Álvarez, 2024).
